# Analysis of Threshold Voltage Shift for Full V_GS_/V_DS_/Oxygen-Content Span under Positive Bias Stress in Bottom-Gate Amorphous InGaZnO Thin-Film Transistors

**DOI:** 10.3390/mi12030327

**Published:** 2021-03-19

**Authors:** Je-Hyuk Kim, Jun Tae Jang, Jong-Ho Bae, Sung-Jin Choi, Dong Myong Kim, Changwook Kim, Yoon Kim, Dae Hwan Kim

**Affiliations:** 1 The School of Electrical Engineering, Kookmin University, Seoul 02707, Korea; jhyuck93@kookmin.ac.kr (J.-H.K.); jtjang@kookmin.ac.kr (J.T.J.); jbae@kookmin.ac.kr (J.-H.B.); sjchoiee@kookmin.ac.kr (S.-J.C.); dmkim@kookmin.ac.kr (D.M.K.); 2 Circadian ICT Research Center, Kookmin University, Seoul 02707, Korea; ncwkim@kookmin.ac.kr; 3 The School of Electrical and Computer Engineering, University of Seoul, Seoul 02504, Korea

**Keywords:** indium-gallium-zinc-oxide thin-film transistors (-IGZO TFT), oxygen content, instability, electron trapping, hole trapping, donor-like state creation

## Abstract

In this study, we analyzed the threshold voltage shift characteristics of bottom-gate amorphous indium-gallium-zinc-oxide (IGZO) thin-film transistors (TFTs) under a wide range of positive stress voltages. We investigated four mechanisms: electron trapping at the gate insulator layer by a vertical electric field, electron trapping at the drain-side GI layer by hot-carrier injection, hole trapping at the source-side etch-stop layer by impact ionization, and donor-like state creation in the drain-side IGZO layer by a lateral electric field. To accurately analyze each mechanism, the local threshold voltages of the source and drain sides were measured by forward and reverse read-out. By using contour maps of the threshold voltage shift, we investigated which mechanism was dominant in various gate and drain stress voltage pairs. In addition, we investigated the effect of the oxygen content of the IGZO layer on the positive stress-induced threshold voltage shift. For oxygen-rich devices and oxygen-poor devices, the threshold voltage shift as well as the change in the density of states were analyzed.

## 1. Introduction

Amorphous InGaZnO (a-IGZO) thin-film transistors (TFTs) have made significant progress, particularly in display and mobile electronics, owing to their high mobility, high transparency to visible light, and low process temperatures [1,2,3]. The instability issues of a-IGZO TFTs have recently attracted significant attention. For the reliable incorporation of a-IGZO TFTs into the driving circuits for display application, it is crucial to understand the degradation mechanisms of the device under bias stresses. Many research groups have reported stress-induced instability characteristics [4,5,6,7,8,9,10,11,12,13,14]. Most of these studies have focused on the verification of degradation mechanisms, such as charge trapping, hole trapping, and donor-state creation. However, no studies have been reported that analyze the instability for a wide stress voltage region, including a very large gate or drain voltage. In addition, a thorough understanding of the effect of oxygen content in IGZO thin films on the degradation mechanisms under various stress conditions has not been reported.

In this study, we analyzed the change in threshold voltage (*V*_T_) in a-IGZO TFTs over a stress voltage range of 10–50 V. Local *V*_T_ shifts at the drain and source sides were measured by reverse and forward read-out conditions, respectively. In addition, we investigated the degradation mechanism by extracting the density of states (DOS) before and after stress. Further, the effect of different oxygen contents was analyzed by adjusting the oxygen flow rate (OFR) in the deposition process of the IGZO layer on the positive stress-induced *V*_T_ shift.

## 2. Device Sample Preparation

**Figure 1:**
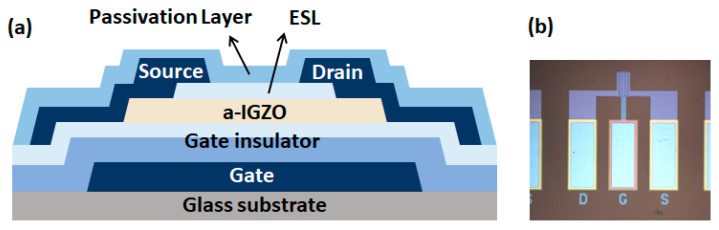
Amorphous InGaZnO (a-IGZO) thin-film transistor Sample: (**a**) Schematic structure of bottom-gate a-IGZO TFT device; (**b**) Top view of layout image.

Figure 1 shows a schematic diagram of the bottom-gate a-IGZO TFT device structure. The width (*W*_ch_) and length of the device channel (*L*_ch_) were 100 and 15 μm, respectively.

Key fabrication steps of the device are as follows:

The molybdenum (Mo) bottom gate was patterned on a glass substrate by room temperature (RT) sputtering. The gate insulator (GI) was formed with SiN_x_ and SiO_2_ using plasma-enhanced chemical vapor deposition (PE-CVD) on the bottom gate. The equivalent oxide thickness (EOT) of GI is 258 nm. The a-IGZO layer with a thickness of 50 nm was sputtered in an oxygen-argon atmosphere. Different devices—oxygen-poor (O-poor) and oxygen-rich (O-rich)—are fabricated in different OFRs by direct current (DC) sputtering at RT. O_2_ flow rates of 21 and 63 sccm were used for the O-poor and O-rich devices, respectively. An Ar flow rate of 25 sccm was used for both the devices. After the deposition of the IGZO layer, the SiO_x_ etch-stop layer (ESL) and source and drain electrodes were patterned with Mo. Finally, SiO_x_ and SiN_x_ passivation layers were deposited, followed by annealing at 250 °C.

The electrical properties of the fabricated devices were measured in the dark at room temperature using an Agilent B1500A semiconductor parameter analyzer (Agilent Technologies, Santa Clara, CA, United States).

## 3. Results and Discussion

**Figure 2:**
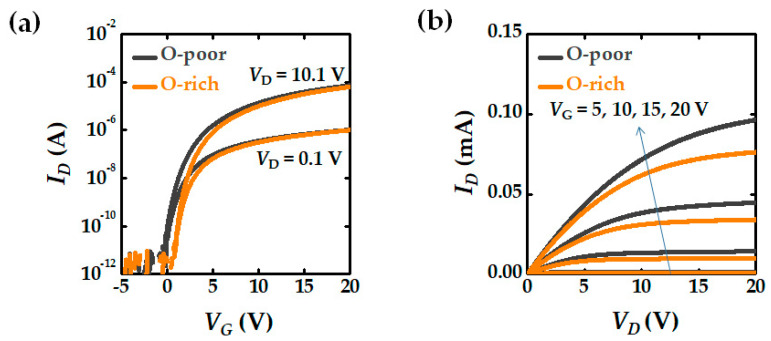
Current-voltage characteristics of IGZO TFT device: (**a**) Transfer characteristics of O-poor and O-rich devices; (**b**) Output characteristics of O-poor and O-rich devices.

Mechanisms of stress-induced *V*_T_ shift in the fabricated a-IGZO TFTs were verified using I-V characteristics and monochromatic photonic C-V (MPCV) sub-gap DOS measurements [15,16,17,18,19]. Figure 2 indicates the transfer characteristics and output characteristics of pristine a-IGZO TFTs. After applying 10 s of stress at 25 different bias conditions with *V*_GS_ and *V*_DS_ bias matrices in the range of 10–50 V, contour of *V*_T_ shift (Δ*V*_T_) is represented. A constant current method with a constant drain current (*I*_D_ = 10^−8^ A) was used to extract the *V*_T_. To observe the *V*_T_ shift in the source and drain regions separately, a reverse and forward readout condition was used. In general, the threshold voltage of a transistor is determined by the region at which the barrier potential is high within the channel region. Since the barrier potential of the channel is low in the drain region where a relatively high voltage is applied, the threshold voltage of the transistor is determined not by the drain region, but by the source region where a relative low voltage is applied. Therefore, Forward read-out can measure source-side *V*_T_ by applying a higher bias at the drain side, and reverse read-out can measure drain-side *V*_T_ by switching the source and drain electrodes. Note that the forward read-out or reverse read-out measurements does not cause a *V*_T_ shift.

### 3.1. V_T_ Shift Mechanisms

**Figure 3:**
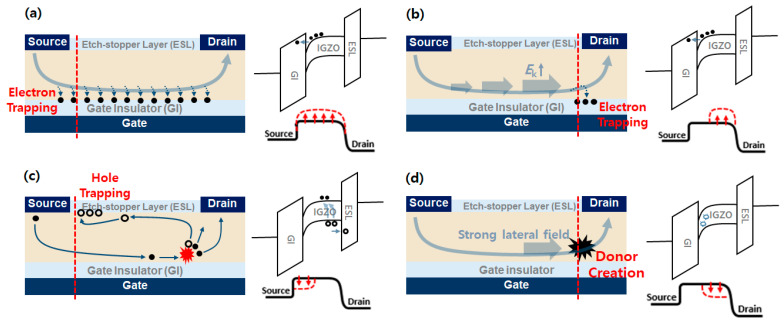
Schematic structures and energy band diagrams of four different mechanisms of *V*_T_ shift in IGZO TFT device: (**a**) Electron trapping at the GI layer by vertical electric field; (**b**) Electron trapping at the drain-side GI layer by HCI; (**c**) Hole trapping at the source-side ESL by impact ionization; (**d**) Donor-like state creation in the drain-side IGZO layer by lateral electric field.

As shown in Figure 3, *V*_T_ shift can occur by four different mechanisms in a-IGZO TFTs, as follows:

1. Electron trapping in the interface of bulk GI layer occurs by a vertical electric field (positive gate voltage), as shown in Figure 3a. This occurs throughout the entire channel area. However, when a high voltage is applied to the drain, it occurs more at the source-side interface because *V*_GS_ > *V*_GD_. In this case, a positive *V*_T_ shift occurs owing to the screen effect of trapped electrons [6,20,21].
2. Electron trapping at the drain-side GI layer occurs by hot-carrier injection (HCI), as shown in Figure 3b. HCI is one of the mechanisms that adversely affects the reliability of semiconductors. Electrons gain sufficient kinetic energy by a large lateral electric field to overcome a potential barrier between the IGZO channel and the GI [22]. These electrons can be trapped in the GI and also cause a positive *V*_T_ shift of the device [13,23].
3. Hole trapping at the source-side ESL occurs via impact ionization, as shown in Figure 3c. This is also caused by the lateral electric field because of the highly applied drain bias, and electrons can get enough energy to give rise to impact ionization, and an electron-hole pair (EHP) is finally generated. The generated holes are trapped at the interface between the IGZO channel and ESL while drifting to the source side because of the absence of a path to exit (body electrode), which causes a potential increase in the channel, thus resulting in a negative *V*_T_ shift [13].
4. Donor-like state creation in the drain-side IGZO layer occurs by a lateral electric field, as shown in Figure 3d. In general, oxygen vacancies in a-IGZO were found to act mostly as shallow donors. In some cases, a deep donor can be created through the creation of a metal-metal bond with an electronic property able to capture two electrons. Donor-like states originate from the impact ionization of oxygen vacancies in the IGZO layer (Vo + e^−^ → Vo^2+^ + 3e^−^). These positively charged states result in a negative *V*_T_ shift [13,24,25,26,27]. This mechanism is dominant near the drain side because the lateral electric field is higher than the source side.

### 3.2. V_T_ Shift Results

**Figure 4:**
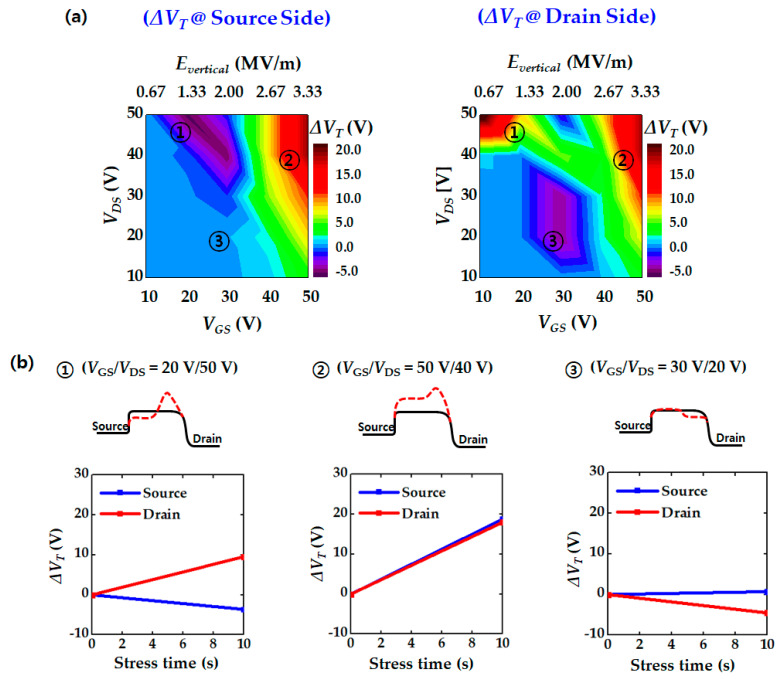
*V*_T_ shift results after various stress bias (O-poor device case): (**a**) Contour maps of *V*_T_ shift at source and drain side (forward and reverse read-out); (**b**) *V*_T_ change as a function of stress time at three major bias regions with schematic illustration of barrier height modulation.

Figure 4a shows *V*_T_ shift contour maps at the source and drain sides under various stress conditions (*V*_GS_ and *V*_DS_ biases). The *V*_T_ change was observed after applying a stress bias for 10 s to the O-poor devices. This contour map helps to easily identify the *V*_T_ instability characteristics for various stress bias ranges. Interestingly, three stress-bias regions with different *V*_T_ shift trends were observed.

In the bias region of ① with relatively low *V*_GS_ and high *V*_DS_ stress (*V*_GS_ = ~20 V and *V*_DS_ = ~50 V), a very high lateral electric field is applied; thus, impact ionization and HCI are the dominant mechanisms. Hole trapping at the source side occurs from EHP generation due to impact ionization and results in a negative *V*_T_ shift. On the other hand, electron trapping due to HCI causes a positive *V*_T_ shift at the drain side. 

In the bias region of ②, a very high voltage was applied to the gate (*V*_GS_ = ~50 V). When a large vertical electric field is applied, electron trapping occurs in the entire GI region due to electron tunneling; thus, positive *V*_T_ shifts were observed at both sides. In addition, as the drain stress bias increased, HCI was generated. Consequently, more electron trapping and a larger *V*_T_ shift occurred.

Next, the bias condition of ③ with moderate stress voltages (*V*_GS_ = ~30 V and *V*_DS_ = ~20 V) causes different *V*_T_ shifts at the source and drain sides. A slightly negative *V*_T_ shift occurred at the drain side, while almost no *V*_T_ shift occurred at the source side. Under this stress condition, the creation of donor-like states near the drain side is a dominant *V*_T_-shift mechanism. 

Figure 4b shows the change in *V*_T_ according to the stress time in each bias region. Schematic illustrations of the potential barrier modulation are also presented.

### 3.3. Effect of OFR on V_T_ Instability

**Figure 5:**
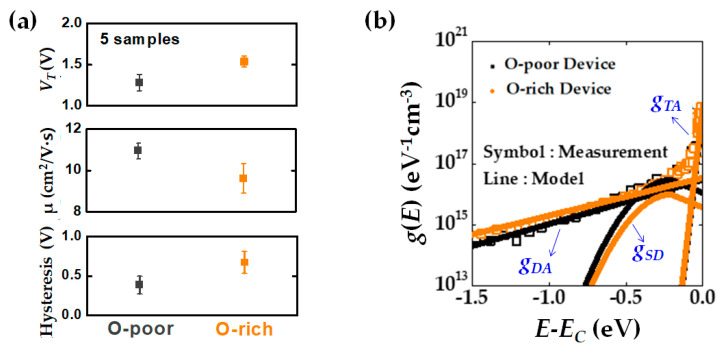
Characteristic comparisons between O-poor and O-rich a-IGZO devices: (**a**) Electrical characteristics (threshold voltage, mobility, and hysteresis) of O-poor and O-rich devices; (**b**) Experimentally extracted DOS distributions of two devices by using MPCV method. Black and orange symbols indicate O-poor and O-rich device, respectively.

Next, the effect of OFR on the electrical characteristics of the a-IGZO TFT devices was observed. Figure 5a shows the pristine electrical characteristics of the O-poor and O-rich devices. 

A high oxygen content results in a higher *V*_T_. This is because the number of oxygen vacancies, which act as shallow donors, decreases. As the oxygen content increases, the number of electron traps generated by the diffusion of oxygen into the GI increases [28,29,30]. Consequently, devices with a higher OFR have lower carrier mobility and a low mean free path. In addition, O-rich devices cause greater hysteresis than O-poor devices. Here, the hysteresis is defined as the difference in *V*_T_ between the *V*_GS_ sweep from 20 to −20 V and that from −20 to 20 V. Large hysteresis indicates many electrons trapped in the GI and GI/IGZO interfaces. 

The experimentally extracted DOS in the IGZO layers are shown in Figure 5b. The DOS was extracted using the MPCV measurement method based on the photoresponsive C-V characteristics in the subgap energy range (*E_V_* < *E* < *E_C_*). The detailed methodology was described in our previous work [17]. Capacitance was measured by a LCR meter (HP4284A) using a 50 kHz AC signal. A 5 mW illumination source with a photon energy of 2.8 eV was used. The extracted DOS (*g*(*E*)) near the conduction band were modeled to be a superposition of three components: 1. shallow donor-like states (*g_SD_*), 2. acceptor-like deep states (*g_DA_*), 3. acceptor-like tail states (*g_TA_*). Each DOS was modeled according to Equation (1):

(1) $$\begin{array}{c} g\left(E\right)=g_{TA}\left(E\right)+g_{DA}\left(E\right)+g_{SD}\left(E\right)\\ =N_{TA}\exp\left(-\frac{E_{C}-E}{kT_{TA}}\right)+N_{DA}\exp\left(-\frac{E_{C}-E}{kT_{DA}}\right)+N_{SD}\exp\left(-{\left(\frac{E_{C}-E-E_{SD}}{kT_{SD}}\right)}^{2}\right) \end{array}$$

where *E_SD_* denotes a center energy of the Gaussian peak. 

The O-rich device has a higher DOS near the conduction band edge (*E_C_*), which means that they have more electron traps, as mentioned above. 

**Figure 6:**
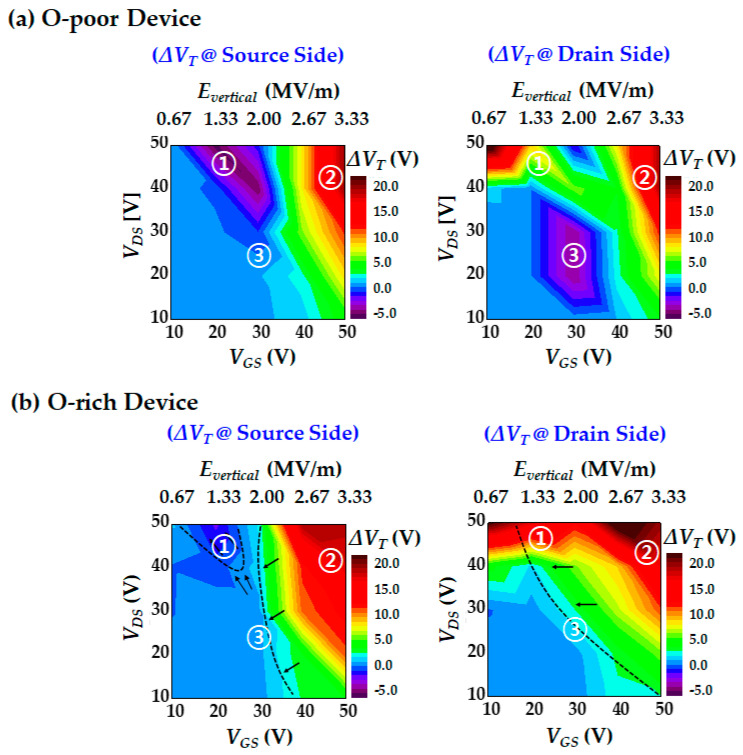
*V*_T_ shift results after various stress bias: (**a**) O-poor device characteristic; (**b**) O-rich device characteristic.

Figure 6 shows the contour maps of the *V*_T_ shift after 10 s of stress applied to the gate and drain electrodes. The two graphs in Figure 6a are Δ*V*_T_ of the O-poor device and the others in Figure 6b are those of the O-rich device. The left graphs are Δ*V*_T_ at the source side, and the right graphs are those at the drain side. Compared with the O-poor device, the O-rich device exhibited the following changes in each stress bias region (①, ② and ③).

In the O-rich device, hole trapping at the source side and electron trapping at the drain side caused by impact ionization in the bias region of ① are reduced. This can be interpreted as electrons of the O-rich device struggle to obtain sufficient kinetic energy to cause impact ionization due to poor carrier mobility and small mean free path.

In addition, O-rich devices have more traps inside the bulk GI and at the interface of GI/IGZO, so more electrons can be trapped for the same gate stress voltage. Thus, the bias region of ②, where the electron trapping by the vertical electric field is the dominant mechanism, is extended, as can be seen in Figure 6b. In addition, it should be noted that the self-heating effect also affects the electron density trapped at the interface and bulk GI layer. Under the same operation voltages, the drain current of the O-poor device is larger than that of the O-rich device as can be seen in Figure 2, and thus the Joule heating is also larger. Consequently, the O-poor device generates more self-heating-assisted e-trapping [30]. Nevertheless, the fact that the *V*_T_ shift of the O-rich devices is larger than that of the O-poor device indicates that the mechanism of electron trapping at the GI layer by the vertical electric field is dominated by the trap density rather than by the self-heating effect.

Finally, in the case of the O-rich device, the donor-like state creation at the drain side under the bias region of ③ is significantly reduced. This result can be interpreted in a similar way to the bias region of ①. As mentioned earlier, the O-rich device has lower carrier mobility and smaller mean free path. Consequently, the number of electrons that obtain sufficient kinetic energy to cause impact ionization of oxygen vacancies in the O-rich device is smaller than that in the O-poor.

**Figure 7:**
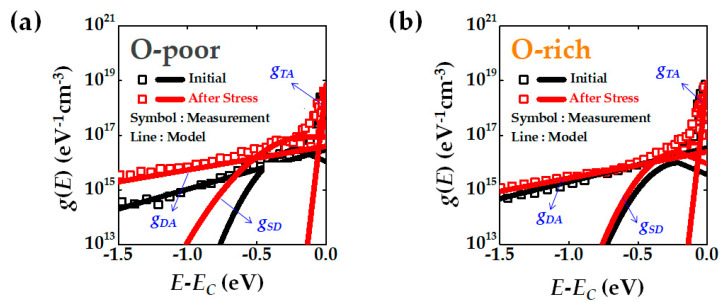
Extracted DOS distributions before and after stress: (**a**) The DOS change of O-poor device; (**b**) The DOS change of O-rich device.

To further analyze this, DOS was extracted before and after applying the stress bias of region ③ (*V*_GS_ = 30 V and *V*_DS_ = 20 V), as shown in Figure 7. The O-poor device exhibits an increase in the donor-like state(*g*_SD_) after stress, whereas the increase in the donor-like state of the O-rich device is relatively small, as seen in Figure 7b.

## 4. Conclusions

In this study, the characteristics of the *V*_T_ shift caused by various positive stress voltages were investigated. For various gate and drain stress voltage pairs (*V*_DS_/*V*_DS_ = 10~50 V), local *V*_T_s at the source and drain sides were observed. In particular, we classified three stress bias regions using contour maps of the *V*_T_ shift. In the bias region with relatively low *V*_GS_ and high *V*_DS_ stress, electron trapping at the drain-side GI layer, and hole trapping at the source-side ESL are the dominant mechanisms. In the bias region under a high gate voltage, electron trapping predominately occurs at the GI layer by the vertical electric field. In the bias region with moderate stress voltages (*V*_GS_ = ~30 V and *V*_DS_ = ~20 V), donor-like state creation in the drain-side IGZO layer is the dominant mechanism. In addition, we analyzed the effect of the oxygen content of the IGZO layer on each *V*_T_ shift mechanism. Our instability analysis for a wide stress voltage can provide useful information when determining deterioration acceleration conditions for the purpose of reliability testing or when dealing with new applications that require a large operation voltage.
